# Periictal water drinking revisited: Occurrence and lateralizing value in surgically confirmed patients with focal epilepsy

**DOI:** 10.1002/epi4.12690

**Published:** 2023-01-29

**Authors:** Yuhei Tanno, Takashi Matsudaira, Naotaka Usui, Hiroshi Ogawa, Kentaro Tokumoto, Norihiko Kawaguchi, Akihiko Kondo, Takuji Nishida, Yukitoshi Takahashi

**Affiliations:** ^1^ National Epilepsy Center, NHO Shizuoka Institute of Epilepsy and Neurological Disorders Shizuoka Japan

**Keywords:** autonomic behavior, focal epilepsy, language dominant hemisphere, lateralizing value, periictal water drinking

## Abstract

**Objective:**

Periictal water drinking (PIWD), which is a rare seizure‐related autonomic behavior, has been reported in temporal lobe epilepsy (TLE) but only rarely in extra‐TLE. Additionally, the lateralizing value of PIWD is controversial. We aimed to clarify the occurrence and lateralizing value of PIWD in patients with focal epilepsy.

**Methods:**

This retrospective study included 240 focal epilepsy patients aged >10 years with a favorable postoperative seizure outcome (*Engel* class I). PIWD was defined as water drinking behavior during a seizure or within 2 min in the postictal phase. The occurrence of PIWD documented on video‐electroencephalogram monitoring was assessed. The lateralizing value of PIWD was analyzed among patients whose language dominant hemisphere was identified.

**Results:**

Twenty‐three (9.5%) patients exhibited PIWD. PIWD occurred more frequently in frontal lobe epilepsy (FLE; eight of 41 patients, 19.5%) than in TLE (15 of 188 patients, 8%). The occurrence of PIWD was significantly different between FLE and extra‐FLE (*P* = 0.035), with a low positive predictive value (34.8%). In FLE with PIWD, all but one patient underwent resective surgery involving the medial frontal lobe. In 194 patients whose language dominant hemisphere was determined, the lateralizing value of PIWD in FLE and TLE showed no statistical significance (*P* = 0.69 and *P* = 0.27, respectively).

**Significance:**

Periictal water drinking occurred more often in FLE than TLE. Thus, PIWD might not be a specific periictal symptom in TLE. There was no evidence for the lateralizing value of PIWD in FLE and TLE. These findings can provide useful clinical clues for preoperative evaluations to estimate the epileptogenic zone based on seizure semiology and allow for a better understanding of pathophysiological insights into PIWD.

**Key Points**

- Periictal water drinking (PIWD), a seizure‐related autonomic behavior, is reported mainly in patients with temporal lobe epilepsy (TLE)
- The occurrence of PIWD in extra‐TLE is rarely described, and the lateralizing value of PIWD is under debate
- In our study, PIWD occurred more often in frontal lobe epilepsy (FLE) than TLE; PIWD had no significant lateralizing value in FLE and TLE
- PIWD might not be a specific periictal symptom in TLE and is most likely not a reliable lateralizing sign
- These findings can be useful in preoperative evaluation based on seizure semiology and may also enhance the understanding of PIWD pathophysiology

## 1 INTRODUCTION

Periictal seizure symptoms provide important clues regarding localization and lateralization for estimating the epileptogenic zone (EZ) during presurgical evaluation. A variety of periictal autonomic behaviors including nose wiping, coughing, and spitting have been reported in both adult and childhood epilepsy.^1^, ^2^, ^3^ These behaviors tend to appear natural and similar to behaviors that occur during common daily activities or may even be too subtle; they are thus usually not identified as seizure‐related behaviors. Certain periictal autonomic behaviors show localizing and lateralizing values, reflecting the paroxysmal disturbance of the central autonomic system (CAS).^2^, ^3^ These behaviors can provide further clues into the mechanism and propagation pathways underlying the neural networks and substrates of the CAS involved in epileptic seizures.^1^

Periictal water drinking (PIWD), which is a rare seizure‐related autonomic behavior, has been described as a characteristic drinking behavior exhibited during the ictal or postictal phases associated with electroclinical seizures.^4^, ^5^, ^6^, ^7^, ^8^ PIWD is commonly reported in patients with temporal lobe epilepsy (TLE); however, the occurrence of PIWD in extra‐TLE has rarely been described.^9^ Several previous studies have suggested that PIWD exhibits a significant lateralizing value in nondominant TLE. These studies proposed that the pathophysiology of PIWD included the propagation of seizures to the hypothalamus and that PIWD might display a nondominant hemispheric representation of the CAS, revealing lateralization.^4^, ^6^ Other studies, in contrast, could not find any evidence for the lateralizing value of PIWD in TLE.^7^, ^8^ Moreover, in some studies, the EZ was not identified from postsurgical seizure outcomes, and the dominant hemisphere was estimated based on the patient's dominant hand. Thus, PIWD has both clinical and theoretical importance. However, whether PIWD occurs in extra‐TLE as well as TLE and whether it exhibits lateralizing value with reference to the nondominant hemisphere are yet unclear.

The aim of our study was to clarify the occurrence and lateralizing value of PIWD and to describe the characteristics of PIWD in patients with focal epilepsy who achieved favorable postoperative seizure outcomes. Our findings may provide further insight into the pathophysiology of PIWD.

## 2 MATERIALS AND METHODS

### 2.1 Participants

We reviewed the records of consecutive patients aged >10 years with drug‐resistant focal epilepsy who underwent evaluations for epilepsy surgery between March 2010 and December 2019 at the NHO Shizuoka Institute of Epilepsy and Neurological Disorders in Japan. We included patients who had undergone resective surgery and excluded those who had undergone palliative surgery (such as corpus callosotomy and vagus nerve stimulation) or surgery that was performed across the cerebral lobes. A total of 370 patients who had undergone epilepsy surgery were identified. Patients who achieved a favorable seizure outcome at least 2 years after surgery (*Engel* class I) were included. A total of 240 patients satisfied the above criteria and were included in this retrospective study. We divided the patients with surgically defined EZ into three groups: EZ on frontal lobe, temporal lobe, and posterior cortex (parietal or occipital lobe). We defined extra‐FLE as EZ on temporal lobe and posterior cortex, and extra‐TLE as EZ on frontal lobe and posterior cortex.

All patients underwent presurgical evaluation including long‐term video‐electroencephalogram (EEG) monitoring, 1.5 or 3 Tesla magnetic resonance imaging (MRI), and single‐photon emission computed tomography (SPECT) and/or [^18^F] fluorodeoxyglucose positron emission tomography. Long‐term video‐EEG monitoring was performed using the EEG‐1000 instrument (Nihon Kohden), and the standard 10–20 system of electrode placement was used in all cases. T1 and T2 or sphenoidal electrodes were added when necessary. In some patients, the Wada test or a functional MRI was undergone to determine the dominant hemisphere for language/speech functions. Additional intracranial electrode was conducted to determine the EZ and the extent of resection when the EZ could not be sufficiently identified and/or in the absence of a clear lesion on MRI after estimating the EZ based on the above evaluations.

This retrospective study was reviewed and approved by the Ethics Committee of the NHO Shizuoka Institute of Epilepsy and Neurological Disorders (2021–28).

### 2.2 Seizure analysis

We reviewed all analyzable seizures recorded by video‐EEG monitoring; the monitoring was performed immediately prior to epilepsy surgery as part of preoperative evaluation. We analyzed all focal awareness seizures and focal impaired awareness seizures documented on video‐EEG monitoring; focal‐to‐bilateral clonic‐tonic seizures were not analyzed. We defined PIWD in a focal impaired awareness seizure as “drinking water behavior” exhibited during a seizure or up to 2 min postictal.^4^ PIWD in focal awareness seizures was defined as “drinking water behavior” within 2 min after the patient was informed of the occurrence of a seizure. In focal impaired awareness seizures, we applied the term ictal phase for the time period during the EEG alteration and the term postictal phase for the time period within 2 min after the end of the EEG alteration. Two authors (Y. Tanno and T.M.) evaluated the seizures individually, and only those instances of PIWD behavior that were acknowledged by both authors were included.

### 2.3 Statistical analysis of the occurrence and lateralizing value of PIWD

We divided patients with PIWD into groups according to the affected cerebral lobe: Frontal and Temporal PIWD groups. The clinical characteristic difference including age at surgery, sex, and age at seizure onset between Frontal and Temporal PIWD groups were also investigated.

We analyzed the difference in the occurrence of PIWD between FLE and extra‐FLE, and TLE and extra‐TLE. We assessed the lateralizing value of PIWD on each cerebral lobe among patients whose language dominant hemisphere had been identified.

The Fisher's exact test and *t* test were used for the statistical evaluation of categorical variables. Statistical significance was set to *P* < 0.05.

### 2.4 Clinical seizure semiology and extent of surgical resection on extra‐TLE

A previous report described the seizure semiology of TLE associated with PIWD,^4^ but the detailed seizure semiology of extra‐TLE with PIWD is unclear. Thus, we assessed the seizure semiology associated with this condition. With reference to pre‐ and postoperative MRI, the approximate extent of visible lesion and of surgical resection area on extra‐TLE were illustrated on a structural MRI scan template (the Montreal Neurological Institute neuroanatomic space; http://www.bic.mni.mcgill.ca) to examine the neuroimaging features of PIWD.

## 3 RESULTS

### 3.1 Occurrence of periictal water drinking

**FIGURE 1:**
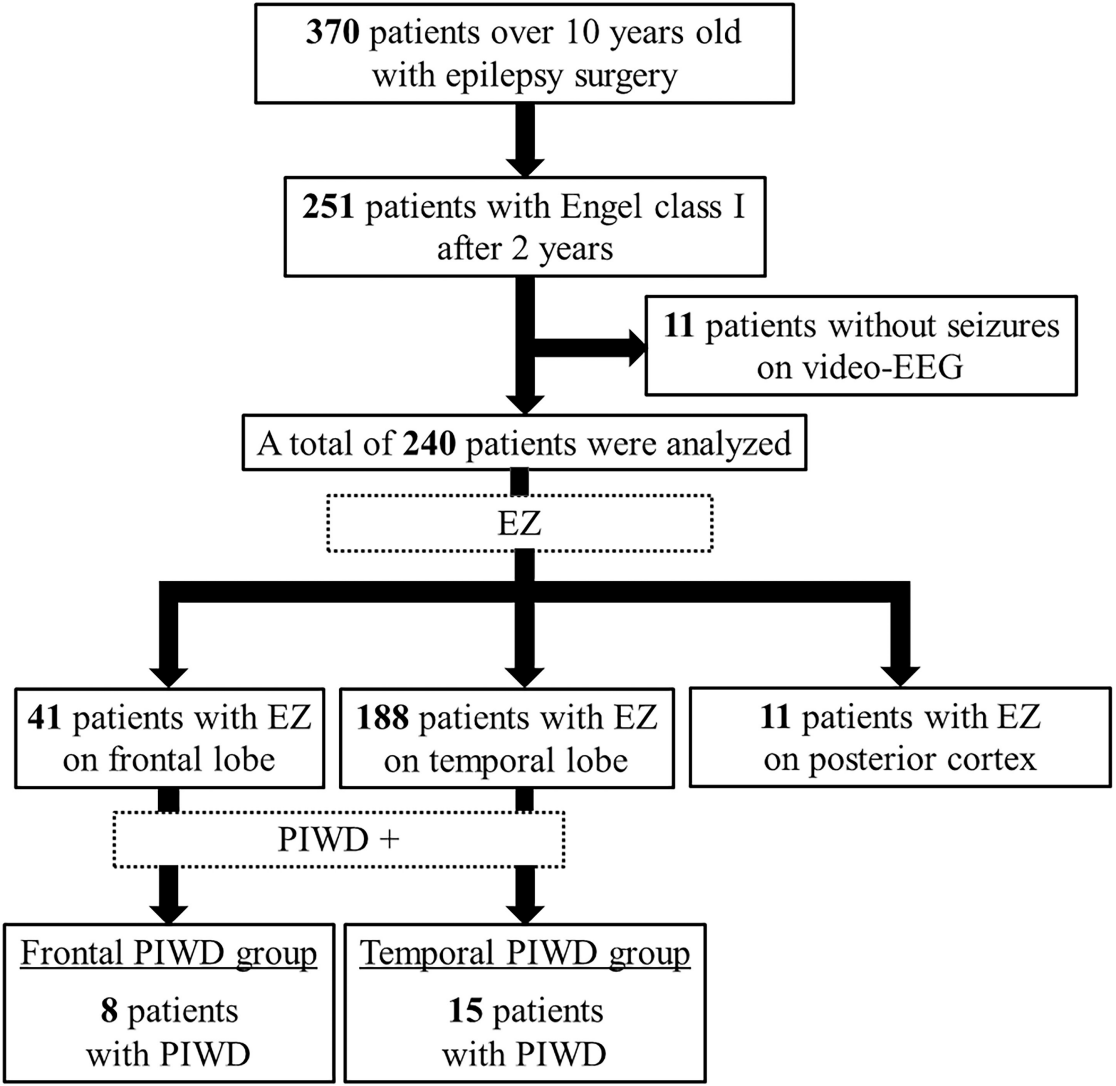
Diagram depicting the number of patients with periictal water drinking behavior and with favorable postsurgical seizure outcome in our retrospective study. Schematic diagram illustrates the number of patients included and excluded in this study. A total of 240 patients with favorable postsurgical seizure outcomes (*Engel* class I) in whom the epileptogenic zone had been identified were analyzed. Periictal water drinking (PIWD) was observed in a total of 23 patients (9.5%): eight of 41 patients (19.5%) in the Frontal PIWD group and 15 of 188 patients (7.9%) in the Temporal PIWD group; none of the 11 patients with posterior cortex (parietal lobe or occipital lobe) epilepsy exhibited PIWD. EEG, electroencephalography, EZ, epileptogenic zone, PIWD, periictal water drinking

A total of 240 patients who achieved *Engel* class I were analyzed: 41 patients with frontal lobe epilepsy (FLE), 188 with TLE, and 11 associated with posterior cortex. Figure 1 presents a flowchart of the patient selection in the study. PIWD occurred during one or more recorded seizures in 23 patients (9.5%): in eight patients (3.3%) with FLE and 15 (6.2%) with TLE; no instances of PIWD were observed among patients with posterior cortex epilepsy. The occurrence of PIWD was more frequent in FLE than in TLE (Frontal PIWD group: 19.5%, Temporal PIWD group: 8%; *P* = 0.04).

The clinical characteristics of the Frontal and Temporal PIWD groups are summarized in Table 1. There was no statistically significant difference in the age at surgery, sex, and age at seizure onset between the groups. All but one patient in the Frontal and Temporal PIWD groups underwent the Wada test to determine the language dominant hemisphere.

**TABLE 1:** The summary of clinical features of patients with periictal water drinking

Clinical characteristic	Frontal PIWD group (n = 8)	Temporal PIWD group (n = 15)	*P value*
Age at surgery, y ± SD	30.6 ± 6.6	39.0 ± 13.8	0.12
Male, No. (%)	8 (100%)	10 (66.7%)	0.12
Age at seizure onset, y	12.5 ± 12.4	15.3 ± 9.8	0.59
Duration of epilepsy, y	18.1 ± 8.4	24.0 ± 14.5	0.31
Frequency of seizures			
Daily–weekly	7	9	
Monthly–yearly	1	6	
Dominant hemisphere^a^, No. (Left/Right/Bilateral)	Left 7, Right 1	Left 13, Bilateral 1, n.a 1	
Side of EZ, No. (Left/right)	Left 2, Right 6	Left 6, Right 9	0.66
Operation method	Lesionectomy 7, frontal lobectomy 1	SAH 12, ATL 2, lesionectomy 1	
Pathology, No.	FCD: type1 1, type 2a 2, type 2b 4, non‐specific finding 1.	MTS 13; Ganglioglioma 1; MTS and scar tissue (atrophic changes) after encephalitis 1.	

Abbreviations: ATL, anterior temporal lobectomy; EZ, epileptogenic zone; FCD, focal cortical dysplasia; MTS, mesial temporal sclerosis; n.a., not available; PIWD, periictal water drinking; SAH, selective amygdalohippocampectomy; SD, standard deviation.

^a^ Determined based on the Wada test.

A total of 86 seizures were recorded in the Frontal PIWD group, and 162 seizures were recorded in the Temporal PIWD group. Water was available at the bedside during long‐term EEG in 66 of 86 seizures (76.7%) in the Frontal PIWD group and in 138 of 162 seizures in the Temporal PIWD group (85.2%). Among the seizures in which water was available at the bedside, the PIWD events were observed in 13 seizures (19.7%) in the Frontal PIWD group and in 28 seizures (20.3%) in the Temporal PIWD group. The number of patients who exhibited two or more instances of PIWD was four (50%) with FLE and six (40%) with TLE. PIWD was observed during the ictal phase in two seizures each in the Frontal PIWD and Temporal PIWD groups. No statistically significant differences in the number of total recorded seizures, PIWD seizures, instances of “available water at the bedside,” and duration of long‐term video‐EEG monitoring were observed between the two groups (Table S1). One representative case each of an FLE and a TLE patient with water drinking behavior accompanied by ictal EEG discharge is depicted in Figure S1; one patient with TLE exhibited water drinking behavior during both the ictal and postictal phases.

### 3.2 Statistical analysis of the occurrence and lateraling value of PIWD

Statistical analysis of the occurrence of PIWD is summarized in Table 2. The occurrence of PIWD differed between the FLE and extra‐FLE cases (19.5% and 7.5%, *P* = 0.035) with a sensitivity of 19.5% and a specificity of 92.5%; the positive predictive value (PPV) was 34.8%. In contrast, the occurrence of PIWD was not significantly different between the TLE and extra‐TLE cases (7.9% and 15.4%, *P* = 0.12) with a sensitivity, 7.9% and a specificity, 84.6%.

**TABLE 2:** Occurrence of periictal water drinking in a total of 240 patients

	Frontal vs extrafrontal lobe			Temporal vs extratemporal lobe		
	Frontal lobe (n = 41)	Extrafrontal lobe (n = 199)	*P* value	Temporal lobe (n = 188)	Extratemporal lobe (n = 52)	*P* value
Number of patients with PIWD occurrence, No. (%)	8 (19.5%)	15 (7.5%)	0.035*	15 (7.9%)	8 (15.4%)	0.12

Abbreviation: PIWD, periictal water drinking.

The dominant hemisphere pertaining to language function was determined in a total of 194 patients (30 patients with FLE and 164 with TLE). Data on the lateralizing value of PIWD in FLE and TLE are shown in Table 3. The EZ was located in the nondominant hemisphere in 5 (16.7%) patients with PIWD in FLE and in 9 (5.5%) patients with PIWD in TLE. There was no significant lateralizing value of PIWD in FLE and TLE of the nondominant hemisphere (*P* = 0.69 and *P* = 0.27, respectively).

**TABLE 3:** Lateralizing value of periictal water drinking with reference to the epileptogenic zone in the frontal and temporal lobes

	Frontal lobe (*n* = 30)^a^ %: EZ located on nondominant side			Temporal lobe (*n* = 164)^b^ %: EZ located on nondominant side		
	Nondominant side	Dominant side or bilateral	*P* value	Nondominant side	Dominant side or bilateral	*P* value
PIWD +	5 (62.5%)	3	0.69	9 (64.3%)	5	0.27
PIWD −	11 (50.0%)	11		70 (46.7%)	80	

Abbreviations: EZ, epileptogenic zone; PIWD, periictal water drinking.

a Total 41 patients; 11 patients without assessment of the dominant side for language function.

^b^ Total 188 patients; 24 patients without assessment of the dominant side for language function.

### 3.3 Clinical seizure semiology and extent of surgical resection of the frontal lobe

**FIGURE 2:**
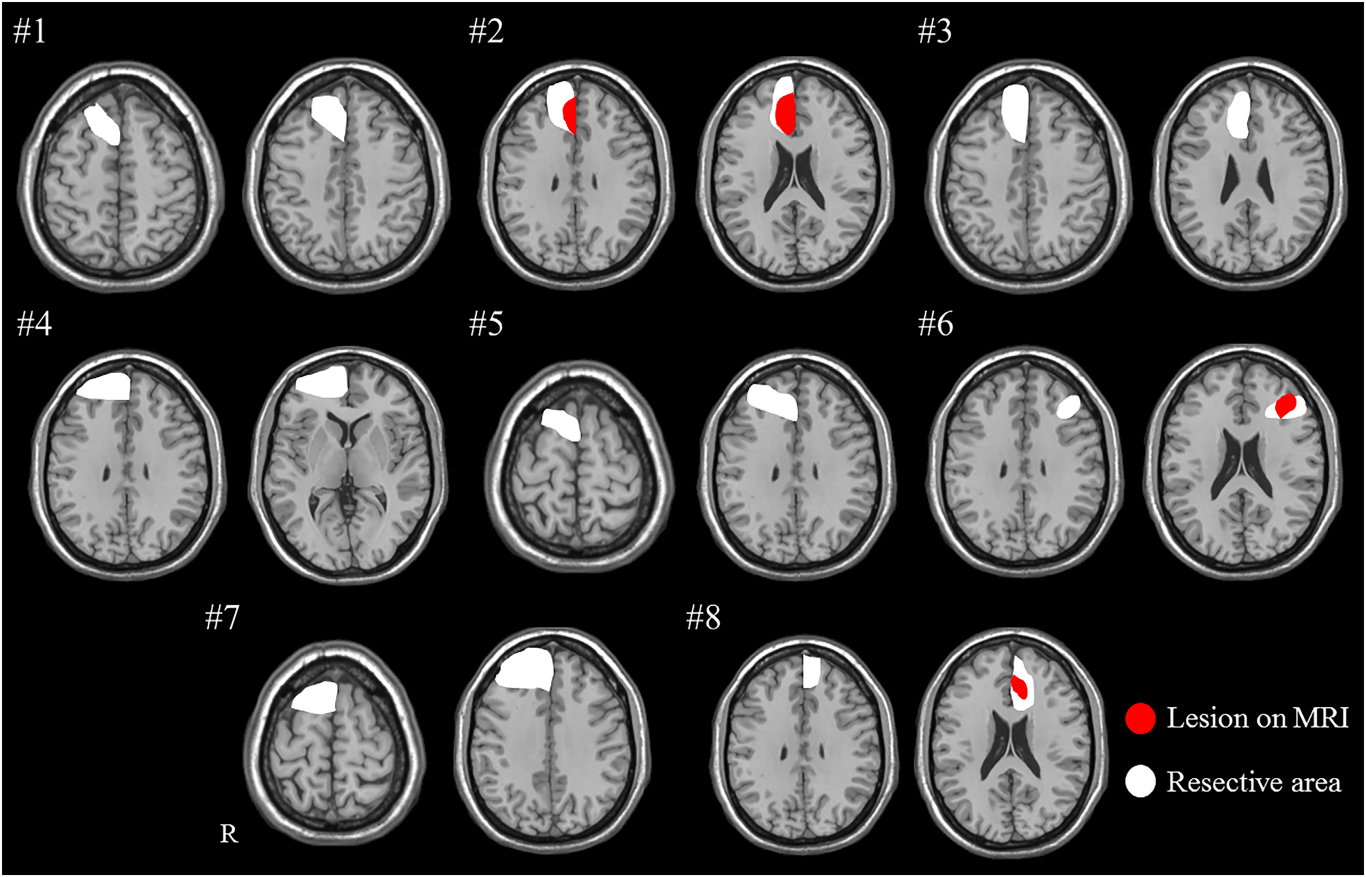
Resection areas and visible lesions are illustrated on a structural magnetic resonance imaging (MRI) scan template in patients with frontal lobe epilepsy who exhibited periictal water drinking behavior. The extent of surgical resection is indicated as a white area, and the visible lesion on MRI is shown as a red area. Three patients (Patient #2, #6, and #8) showed visible lesions on MRI. The resected area involved the right frontal lobe in six patients (Patient #1–#5 and Patient #7) and the left frontal lobe in two patients (Patient #6 and #8). All but one patient underwent resection surgery that involved the medial frontal lobe

The description of the seizure semiology associated with PIWD in FLE is shown in Table 4. None of the patients reported abnormal thirst or compulsive drinking as part of the habitual aura; however, one patient (Patient #8) exhibited ictal speech with the word “water,” which was followed by ictal water drinking. Figure 2 illustrates the approximate extent of visible lesion and the resection area in patients with FLE who exhibited PIWD on an MRI scan template. Three patients showed visible lesions on MRI (#2, 6, and 8); five patients had nonclear lesion (#1, 3, 4, 5, and 7) for whom intracranial EEG was performed to determine the extent of resection area based on presurgical evaluations. All but one patient (Patient #6) underwent resective surgery, which involved the medial frontal lobe: the surgical area extended into the cingulate cortex.

**TABLE 4:** Seizure semiology in frontal lobe epilepsy with periictal water drinking

Patient No.	Ictal status	Seizure classification	Ictal or postictal occurring PIWD	Seizure semiology associated with PIWD	Duration of PIWD from EEG end (s)
#1	Awake	FAS	Postictal	Aura with unpleasant feeling that a seizure is about to occur → flexion of neck and trunk accompanied with twitching of unilateral eyelid and corner of mouth on the left → notifying his seizure → postictal water drinking	16 s after notifying FAS
	Awake	FAS	Postictal	Aura with unpleasant feeling that a seizure is about to occur → notifying his seizure with flexion of body axis → postictal water drinking	6 s after notifying FAS
#2	Sleep	FIAS	Postictal	Opening eyes→ hypermotor seizure with truncal movement → postictal water drinking	3
#3	Sleep	FIAS	Postictal	Repetitive coughing → truncal movement with bilateral facial contraction → gestural automatisms of predominantly the upper extremities → postictal water drinking	45
#4	Sleep	FIAS	Postictal	Short groaning → behavior arrest → Swaying trunk back and forth with hands behind head → oroalimentary automatisms → postictal water drinking	63
#5	Awake	FIAS	Postictal	No aura → behavior arrest → mild gestural automatism of the upper extremities followed by oroalimentary automatisms → postictal water drinking	118
	Sleep	FIAS	Postictal	Opening eyes → gestural automatisms of the upper extremities → postictal water drinking	88
#6	Awake	FIAS	Postictal	Gestural and oroalimentary automatisms → postictal water drinking	56
	Awake	FIAS	Postictal	Gestural and oroalimentary automatisms → Swaying trunk back and forth → postictal water drinking	59
	Awake	FIAS	Postictal	Short behavior arrest → gestural automatisms of both hands → postictal water drinking	24
#7	Awake	FIAS	Ictal	Gestural automatisms → ictal water drinking	‐
#8	Sleep	FIAS	Ictal	Opening eyes → dystonic posturing right upper extremity with hand automatism on left → groaning with paroxysmal up gaze of eyes→ straightening body and crying out → saying “water” → ictal water drinking →behavior arrest	‐
	Sleep	FIAS	Postictal	Opening eyes → dystonic posturing right upper extremity with hand automatism on left → groaning with paroxysmal up gaze of eyes and repetitive beating with right hand → postictal water drinking	66

Abbreviations: EEG, electroencephalogram; FAS, focal awareness seizure; FIAS, focal impaired awareness seizure; PIWD, periictal water drinking.

## 4 DISCUSSION

Our study identified that PIWD occurred in patients with TLE and FLE who achieved *Engel* class I. The occurrence of PIWD has been reported mainly in patients with TLE and varies from 7% to 15.3% in previous reports^4^, ^6^, ^7^, ^8^; Janszky et al.: 7% (total 141 patients); Musilová et al.: 14.4% (14 of 97 patients); Szűcs et al.: 14.5% (eight of 55 patients); and Trinka et al.: 15.3% (10 of 65 patients). These previous reports are in fair agreement with our present study, which included a larger number of patients with TLE. PIWD in extra‐TLE has rarely been investigated; however, one case report described PIWD in a left‐handed boy with left frontal focal status epilepticus.^9^ The present study revealed that PIWD occurred more often in FLE than in TLE, suggesting that PIWD might not be a specific periictal symptom in TLE.

Periictal semiology evolves as the seizure propagates and reflects the part of the cerebral lobe involved in its spread; specific localizing signs can be found based on the above data.^1^, ^2^ Most periictal autonomic symptoms are more prominent in TLE than in FLE, highlighting the important role of the CAS.^1^ Interestingly, our study demonstrated a statistically significant difference in the occurrence of PIWD between FLE and extra‐FLE. However, it is difficult to determine whether the PIWD occurred as a localizing sign of FLE because TLE is more common than FLE. In addition, our results exhibited a relatively low PPV. Thus, it is reasonable to consider that FLE as well as TLE can be a diagnostic clue to estimate the EZ when PIWD occurs.

Periictal water drinking is clinically important because some studies indicate a lateralization sign for the nondominant hemisphere. However, our results showed that PIWD had no lateralizing value in patients with TLE in whom the dominant hemisphere for the language function had been determined. To date, the lateralizing value of PIWD in TLE has been a point of debate.^10^ Some previous studies have proposed that the degree of occurrence of PIWD may indicate its lateralizing value in nondominant TLE,^4^, ^6^ but other studies did not report sufficient supporting evidence to confirm this hypothesis.^7^, ^8^ The major issues with reference to confirming the lateralization value of PIWD in TLE include the rarity of PIWD, the relatively small number of eligible patients, lack of EZ estimation from the postsurgical seizure outcome, and identification of the dominant hemisphere of language functions. Our study included many patients in whom the EZ was identified based on the postsurgical seizure outcome and the dominant hemisphere pertaining to language functions. Our findings imply that PIWD might not represent a lateralizing sign in TLE pertaining to the nondominant hemisphere. In addition, our study could not identify the lateralizing value of PIWD in FLE. To the best of our knowledge, there are no studies on the lateralizing value of PIWD in patients with FLE. Although the relatively small number of patients with FLE implies that the study should be sufficiently powered to establish evidence on lateralization in FLE, PIWD, which was observed in both FLE and TLE, has important implications with reference to the pathophysiology of PIWD.

Various pathophysiological mechanisms associated with water drinking behavior have been reported. In the context of TLE, the pathophysiology underlying PIWD has been discussed with a focus on the hypothalamus, which plays an important role in maintaining tissue water balance.^10^, ^11^ Information from peripheral receptors is processed and integrated in the subfornical organ and hypothalamus and is then conveyed to various cerebral structures.^4^, ^12^ Previous studies have hypothesized that PIWD may be induced by the propagation of epileptic discharges from the mesial temporal lobe to the hypothalamus in patients with TLE.^4^, ^6^ This hypothesis is supported by several studies. A clinical study that employed an implanted depth electrode showed that water drinking was associated with electroclinical seizures starting in the amygdala, hippocampus, and parahippocampal gyrus.^5^ Another study conducted in rats reported that electrical stimulation (especially that of a high frequency) in the lateral hypothalamus (LHA) induced water drinking behavior.^13^, ^14^ Furthermore, activating neurotensin‐expressing LHA neurons specifically promoted drinking behavior, which was induced by an increase in the urge to drink.^15^

Studies using functional imaging revealed that in addition to the hypothalamus, a wide range of cerebral regions are involved in the appearance of thirst and in promoting drinking behavior.^16^, ^17^, ^18^, ^19^, ^20^ One study revealed changes in the regional cerebral blood flow (rCBF) in the anterior cingulate and the insula after the infusion of hypertonic saline.^16^ Another study showed that the regions implicated in inducing thirst included the cingulate cortex, prefrontal cortex, striatum, parahippocampus, and cerebellum. In addition, the correlation between the rCBF levels of the cingulate cortex and insula and the ventral lamina terminalis, which control both thirst and vasopressin secretion, varied according to the states of thirst, indicating that the close functional connectivity of these brain regions is influenced by hydration status.^20^ Thus, the mechanism underlying water drinking may involve the simultaneous activation of both the temporal lobe and frontal lobe regions, especially the cingulate cortex and insula.

Various aspects related to thirst, drinking behavior, and water drinking control associated with water sufficiency have also been investigated. One study indicated that the caudal orbitofrontal cortex was found to be activated after subjects had drunk water to satiety; this region is responsible for the modulation of responses to water based on the physiological state of the body.^19^ Another study demonstrated that postdrinking changes in rCBF in the anterior midcingulate cortex correlated with drinking volumes, despite the fact that thirst‐related activation was found in several cerebral regions.^17^ In addition, the network of regions including the left parietal cortex, left motor cortex, left striatum, left thalamus, and lateral prefrontal cortex in both the hemispheres showed greater activity in the overdrinking condition than in the thirsty condition.^21^ Thus, water drinking control is associated with a wide range of brain regions including the frontal and temporal lobe through the cerebral network.

Our study clarified that PIWD occurred more frequently in FLE; all but one of the patients with FLE underwent resective surgery involving the medial frontal lobe, extending to include the cingulate cortex. Previous reports on the pathophysiology of PIWD revealed that the hypothalamus is one of the important centers that regulates water drinking; the frontal lobe, especially the medial frontal lobe, similarly regulates water drinking activity by regulating thirst and promoting drinking behavior. Therefore, the pathophysiology of PIWD in FLE may be explained as follows: the propagation of epileptic discharges from the frontal lobe to the medial frontal and temporal lobes or the epileptic discharges in the EZ themselves induce thirst, promote drinking behavior, and impair water drinking control, resulting in a higher incidence of PIWD in FLE than in TLE.

The present study has several limitations that must be considered when interpreting the results. The first issue concerns the localization and lateralization value of seizure semiology in posterior cortex epilepsy: the reliability of our results may be limited by the small number of patients with posterior cortex analyzed in the study. The number of patients who receive resective surgery on posterior cortex is generally small. Therefore, a large number of patients need to be studied to clarify the localization and lateralization value of PIWD in posterior cortex epilepsy. The second issue concerns reproducibility; the patients in this study who showed more than two instances of PIWD comprised 50% of the population with FLE and 40% of those with TLE. This indicates that PIWD may not always occur, even in the same patient. However, the specificity of PIWD was relatively high, implying that it may provide valuable clues to estimate the EZ. The third issue is regarding the pathophysiology of PIWD. We could not elucidate the pathophysiology of PIWD as the present study did not examine patients with invasive EEG. Consequently, we were not able to identify a specific cerebral area or the propagation pathway that was associated with PIWD. Thus, applying invasive EEG would be essential in future studies for proving the hypothesis pertaining to PIWD in FLE and TLE as mentioned above.

## 5 CONCLUSION

Our study revealed that PIWD occurred in patients with FLE and TLE without a significant lateralizing value. Although a statistically significant difference of occurrence of PIWD between FLE and extra‐FLE was observed, PIWD may not have localization value in FLE. The pathophysiology of PIWD could be related to the induction of thirst and alterations in drinking behavior and water drinking control, and these may arise from epileptic discharge through the affected cerebral regions in the temporal and frontal lobes.

## AUTHOR CONTRIBUTIONS

Conceptualization and design: T.M., Y. Tanno; Methodology: T.M., Y. Tanno, U.N., A.K.; Formal analysis and investigation: T.M., Y. Tanno, N.U., H.O., K.T., N.K., A.K., T.N., Y. Takahashi; Writing‐original draft preparation: T.M., Y. Tanno; All coauthors provided critical revisions of the manuscript for important intellectual content. The authors read and approved the final manuscript.

## CONFLICT OF INTEREST

None of the authors has any conflict of interest to disclose. We confirm that we have read the Journal's position on issues involved in ethical publication and affirm that this report is consistent with those guidelines.

## ETHICS APPROVAL STATEMENT

This study was reviewed and approved by the Ethics Committee of the Shizuoka Institute of Epilepsy and Neurological Disorders (2021–28).

## PATIENT CONSENT STATEMENT

This retrospective study obtained ethical approval from the Institutional Review Board. All analyzed data were collected as part of routine diagnosis and treatment of each patient, and the clinical data have been anonymized.

## Supporting information


Figure S1

Table S1

## Data Availability

The datasets used and/or analyzed during the current study are available from the corresponding author on reasonable request.
