# Supplementary figures and images for: Periictal water drinking revisited: Occurrence and lateralizing value in surgically confirmed patients with focal epilepsy
Source: Epilepsia Open. 2023 Jan 29;8(1):173–82. doi: 10.1002/epi4.12690 (PMC9977749; doi:10.1002/epi4.12690)

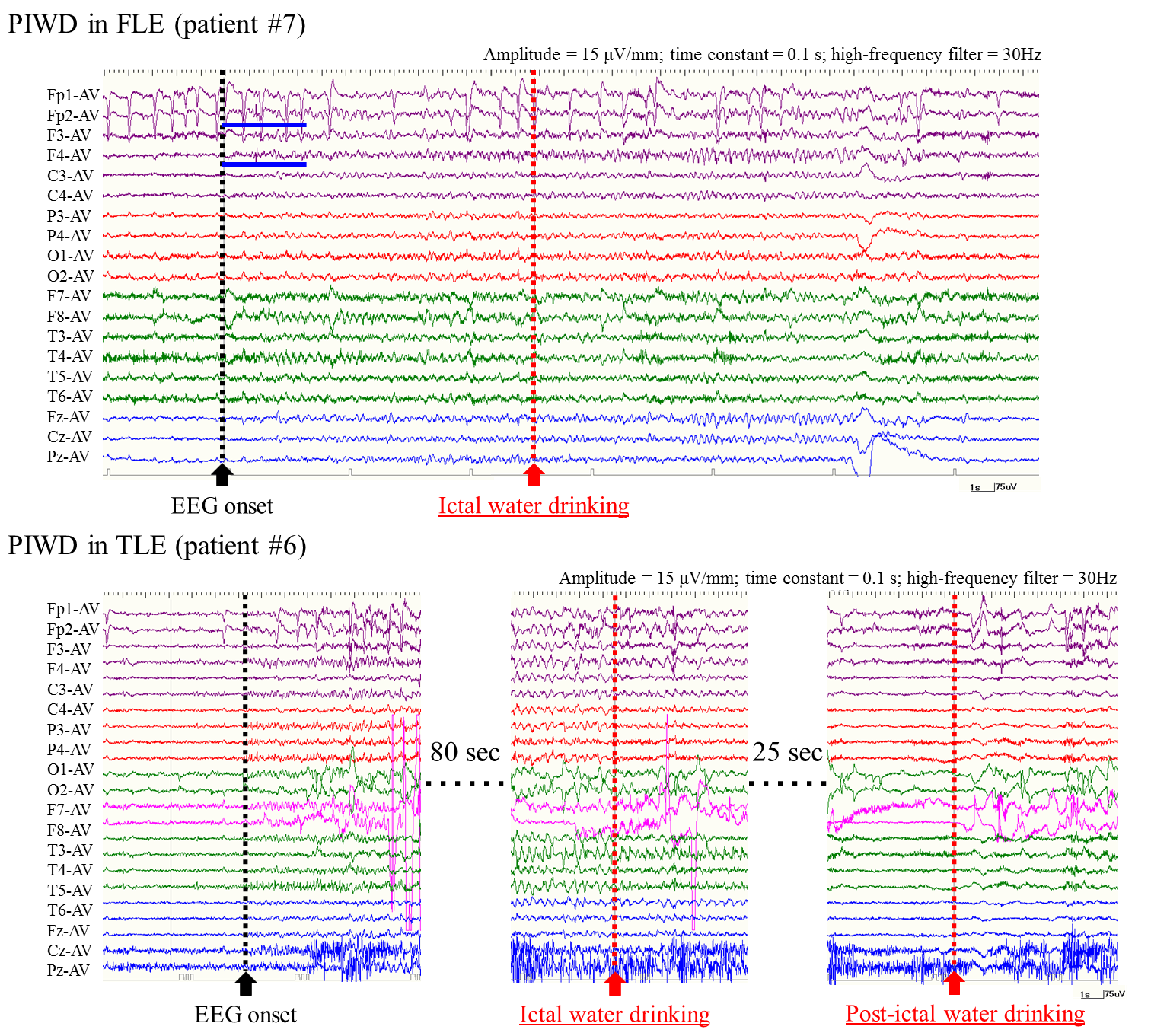

Supplement: Supplementary file 1 — Figure S1 [file EPI4-8-173-s001.tif]
