# Supplementary material for: Periictal water drinking revisited: Occurrence and lateralizing value in surgically confirmed patients with focal epilepsy
Source: Epilepsia Open. 2023 Jan 29;8(1):173–82. doi: 10.1002/epi4.12690 (PMC9977749; doi:10.1002/epi4.12690)
Supplement: Supplementary file 2 — Table S1 [file EPI4-8-173-s002.docx]

| **Supplementary Table 1. Features of the seizures recorded during video-electroencephalography monitoring in patients with peri-ictal water drinking** | | | |
| --- | --- | --- | --- |
|  |  |  |  |
| **Recorded seizure features** | **Frontal PIWD group (n = 8)** | **Temporal PIWD group (n = 15)** | ***p* value** |
| Total recorded seizures, No. | 86 (10.8 ± 5.8) | 162 (10.8 ± 13.4) | 0.99 |
| Number of seizures with PIWD, No. | 13 (1.6 ± 0.7) | 28 (1.9 ± 1.8) | 0.72 |
| Available water at the bedside, No. | 66 (8.3 ± 4.7) | 138 (9.2 ± 12.7) | 0.84 |
| Duration of L-EEG, days | 5.5 ± 2.3 | 6.7 ± 2.1 | 0.21 |
| The status during PIWD (FAS/FIAS), No. | 2/11 | 3/25 | 0.64 |
| Ictal status (Awake/ sleep) | 8/5 | 25/3 | 0.08 |
| PIWD status (Ictal/post-ictal) | 2/11 | 2/27^†^ | 0.58 |
| The duration from EEG end to post-ictal PIWD in patients with FIAS, sec | 58.0 ± 33.4 | 44.1 ±　27.9 | 0.23 |
|  |  |  |  |
| †: One patient showed PIWD during the ictal as well as the post-ictal phase Abbreviations: PIWD, peri-ictal water drinking; EZ, epileptogenic zone; EEG, electroencephalogram; FAS, focal awareness seizure; FIAS, focal impaired awareness seizure; L-EEG, long-term electroencephalography | | | |
